# Environmentally Friendly o–Cresol–Furfural–Formaldehyde Resin as an Alternative to Traditional Phenol–Formaldehyde Resins for Paint Industry

**DOI:** 10.3390/ma17133072

**Published:** 2024-06-22

**Authors:** Marta Depta, Sławomir Napiórkowski, Katarzyna Zielińska, Katarzyna Gębura, Daria Niewolik, Katarzyna Jaszcz

**Affiliations:** 1 Łukasiewicz Research Network—The Institute of Heavy Organic Synthesis “Blachownia”, Energetyków 9, 47-225 Kedzierzyn-Kozle, Poland; marta.depta@icso.lukasiewicz.gov.pl (M.D.); slawomir.napiorkowski@icso.lukasiewicz.gov.pl (S.N.); katarzyna.zielinska@icso.lukasiewicz.gov.pl (K.Z.); katarzyna.gebura@icso.lukasiewicz.gov.pl (K.G.); 2 Department of Physical Chemistry and Technology of Polymers, Silesian University of Technology, M. Strzody 9, 44-100 Gliwice, Poland; daria.niewolik@polsl.pl

**Keywords:** biomass-based resin, o–cresol, furfural, etherification, n-butanol, 2-ethylhexanol

## Abstract

This paper describes studies on the preparation of an o–cresol–furfural–formaldehyde resin in the presence of an alkaline catalyst and its modification with n-butanol or 2-ethylhexanol. The novelty of this research is to obtain a furfural-based resin of the resole type and its etherification. Such resins are not described in the literature and also are not available on the market. The obtained resin based on furfural, which can be obtained from agricultural waste, had a low minimum content of free o–cresol < 1 wt.%, furfural < 0.1 wt.%, and formaldehyde < 0.1 wt.%. The resin structure was characterized by mass spectrometry (ESI-MS), FT-IR, and NMR spectroscopy, which showed the presence of hydroxymethylene groups in the resin before modification and alkyl groups derived from n-butanol and 2-ethylhexanol after modification. The etherified resins had a lower viscosity and were more flexible (DSC) than the resin before modification and they can be used as an environmentally friendly, safe, and sustainable alternative to traditional phenol–formaldehyde resins in the paint industry. They demonstrate the ability to create a protective coating with good adherence to metal substrates and an excellent balance of flexibility and hardness.

## 1. Introduction

Phenol–formaldehyde resins were discovered in 1872 by A. Bayer. They have been on the market since 1907–1909, when Baekeland started their production on an industrial scale. They have a wide range of applications and the ever-increasing demand for these polymer products, with favorable physicochemical properties, is motivating research, leading to the development of new materials showing better performance characteristics than traditional ones [1,2,3,4,5,6,7].

Increasingly stringent EU restrictions on the use and emission of hazardous compounds means that resins containing ≥0.1% unreacted formaldehyde, classified as carcinogenic (cat. 1B) and mutagenic (cat. 2), cannot be commercially marketed. It is therefore advisable to undertake research to completely or partially replace formaldehyde with another aldehyde that is not as harmful to health, e.g., furfural [1,8].

Furfural is considerably less toxic than formaldehyde, and is harmless to the ozone layer. The compound is often referred to as ”gold from rubbish”, as it is obtained from waste biomass (deciduous trees, sugarcane, maize, oat hulls). This offers great prospects for obtaining furfural-based phenolic resins as a possible biomass-sourced alternative to formaldehyde [1,9]. Stenhaus is considered to be the first to develop a furfural-based furan resin, developed in 1840, with the first industrial production starting in 1922. In contrast, phenol–aldehyde resins based on furfural have been produced since the 1920s [10]. However, their popularity and market share was not very significant until the end of the 20th century, when they began to experience a renaissance for environmental reasons [11]. Phenol–furfural resins may become an alternative to phenol–formaldehyde resins in the future, due to the partial or complete replacement of toxic formaldehyde [1] by furfural, which is less toxic and can be obtained from agricultural waste. The need for protection of health and the environment, and the rational use of biomass-based, renewable raw materials for the sake of the environment is a driving force in efforts to completely replace formaldehyde with another aldehyde [8,11].

Phenol–furfural resins have good thermal and chemical resistance and very good dielectric properties, similar to phenol–formaldehyde ones. Compared with phenol–formaldehyde resins, they have better solubility characteristics, and after curing, yield products with less brittleness, which favors their use in the paint industry and for impregnating paper or fabrics for laminating [3,12]. Moreover, their excellent adhesion capabilities onto metals and other materials make them highly suitable for making protective coatings [1]. Despite these advantages, phenol–furfural resins are not widely used. One reason is certainly that the synthesis of these resins is much more difficult and demanding compared to traditional phenolic resins. Due to the lower reactivity of furfural, they are usually obtained at a temperature above 100 °C, using a large amount of basic catalyst and requiring longer reaction times [13,14]. Acid-catalyzed polycondensation of phenol and furfural becomes uncontrolled because of auto polymerization of furfural, which leads to crosslinked products [13].

The main focus of research in the field of phenol–furfural resins has been to improve their qualities. The method showing the greatest progress in recent years has been their modification, i.e., the use of additives with beneficial effects on specific application properties. Modification occurs mainly at the stage of processing of the finished product to improve its specific properties [3,15,16,17,18,19,20,21,22,23,24].

One way of modifying reactive phenol–formaldehyde resins, of the resole type, is through an alkoxylation process, which involves the etherification of hydroxymethylene groups present in the resin, with various types of alcohols (n-butanol, 1,6-hexanediol, ethyleneglycol, polyetherols, 1,2-ethanediol, 2,2-oxydiethanol and 1,4-butanediol, 1-octanol). This modification method is used, among others, in the preparation of resins for varnish coatings [25]. Hydroxymethylene groups in phenolic resins can be easily etherified with alcohol, as they tend to form hydroxybenzyl carbonate ions. To avoid self-condensation reactions, highly hydroxymethylated phenols and an excess of alcohol are used. The etherification reaction is carried out at pH 5–7 and at 100–120 °C. The etherified resoles show improved solubility in aromatic solvents and increased flexibility. They are mainly used as film-forming substances in lacquer products or as impregnating resins for laminates. After etherification, the reactivity of these resins during the curing process is reduced [26]. Some polyhydroxy compounds, such as ethylene glycol, are recommended and used to make phenol–formaldehyde resins more flexible, as seen in a Japanese patent described by Zmihorska-Gotfryd [25]. Using this technique, it is possible to obtain resins with a high dry weight content and low free formaldehyde content, which produce flexible protective coatings.

In the available literature, no attempts to modify phenol–furfural resins by etherification are described. Such resins are also not available on the market. Therefore, it seems appropriate to undertake research into the preparation of resole-type phenol–furfural resins containing reactive hydroxyl groups and their modification. The aim of this study was to develop a method of obtaining an etherified phenol–furfural resin, which could provide an alternative to phenol–formaldehyde resins in paint applications. In the research carried out, phenol was replaced by o–cresol. The new product has the potential to be a sustainable and environmentally friendly alternative to traditional phenol–formaldehyde resins, due to the use of more friendly raw materials.

## 2. Materials and Methods

### 2.1. Chemicals

o–cresol and furfural of ≥99% purity were purchased from Sigma-Aldrich (St. Louis, MO, USA). Formalin with a concentration of 48.5–50.2% was purchased from Lerg S.A. (Pustków, Poland). Basic catalysts used were sodium hydroxide, potassium hydroxide, sodium carbonate, and potassium carbonate, which were purchased from the company P.P.H “STANLAB”Sp.J. (Lublin, Poland). Alcohols used were n-butanol and 2-ethylhexanol of purity ≥99% purchased from GrupaAzotyKędzierzyn-Koźle (Kędzierzyn-Koźle, Poland). Acid catalysts used were malonic acid, oxalic acid, adipic acid, succinic acid, and sebacic acid of purity ≥99%, which were purchased from Sigma-Aldrich.

### 2.2. Synthesis and Apparatus

#### 2.2.1. Synthesis of o–Cresol–Furfural Resin

The reaction vessel was a 1000 cm^3^ round-bottomed flask with a heating bowl, mechanical stirrer, a reflux condenser, and a thermocouple connected to an Almemo temperature gauge and a raw material feed port. The o–cresol and furfural were added at a molar ratio of 1:1.2–2 in the presence of the base catalyst KOH 8–20 g solution 10%. The reaction temperature was 115–150 °C with the reaction times ranging from 10–80 h. An unmodified o–cresol–furfural resin was obtained.

#### 2.2.2. Synthesis of o–Cresol–Furfural–Formaldehyde Resins

The reaction vessel setup was the same as in Section 2.2.1. The o–cresol (K) and furfural(F) were added at a molar ratio of 1:0.5–0.8 in the presence of an alkaline catalyst 8–20 g solution 10%. The reaction temperature was 115–130 °C with a reaction time of 10–12 h. After the first condensation step, 46–50% formalin was introduced in an amount of 0.5–0.8 mole formaldehyde and the reaction was carried out at 80–90 °C for 1–3 h. An unmodified o–cresol–furfural–formaldehyde resin was obtained.

FT-IR (KBr): 3400 cm^−^^1^ (ν, O–H groups); 3075, 1600, 1500 cm^−^^1^ (ɣ, =C–H and C=C in aromatics); 2920, 2877 cm^−^^1^ (ɣ, C–H in –CH_2_ groups); 1484 cm^−^^1^ (δ, C–H in–CH_2_ groups); 1209 cm^−^^1^ (ɣ C–O and δO–H in alcohols and phenols); 1115–879 cm^−^^1^ (δ, C–H in substituted aromatics), 800–700 cm^−^^1^ (δ, C–H out-of-plane in substituted aromatics).

^1^H NMR (600 MHz, (CD_3_)_2_CO, δ): 7.68–7.25 (C_10_–H); 7.25–6.50 (C_3_–H; C_5_–H; C_16_–H; C_18_-H); 6.45–6.29 (C_11_–H; C_12_–H); 6.29–5.68 (C_11_–H; C_12_–H); 4.83–4.68 (C_20_–Ha,b); 4.52–4.37 (K–CH_2_–O–CH2-K); 3.70–3.57 (F–CH_2_–O–, F–CH_2_–O–H); 2.56–2.08 (C_7_–H_3_, C_19_–H_3_, C_8_–H).

^13^C NMR (150 MHz, (CD_3_)_2_CO, δ): 171.23 (C_10_–H); 159.60, 153.69, 153.64 (–C– from furfural); 161.76, 160.29, 160.34, 159.43, 159.36, 158.70, 156.98, 156.86, 156.31, 156.20, 154.74, 154.66, 152.03, 150.80, 149.27 (C–3, C–5, C–16, C–18); 146.83 (C–1, C–2, C–4, C–6, C–13, C–14, C–15, C–16); 144.11, 144.01 (C–3, C–5, C–16, C–18); 139.66 (C–11, C–12); 137.62–136.80 (C–11, C–12); 93.70–92.97 (F–CH_2_–O–, F–CH_2_–O–H); 92.40, 92.30 (C–20); 85.85–85.72 (K–CH_2_–O–CH_2_–K); 45.75–45.20, 44.80–44.40, 43.98–43.79 (C–8); 30.46–29.35 (C–7, C–19, K–CH_2_–K).

#### 2.2.3. Modification of o–Cresol–Furfural–Formaldehyde Resins in Etherification Reaction

The etherification reaction of unmodified o–cresol–furfural–formaldehyde resins was carried out, in the same laboratory apparatus, in the presence of n-butanol or 2-ethylhexanol in an acidic medium at pH = 5–2 at a reaction temperature of 110 °C for 10–15 h by azeotropic distillation. The flask was additionally equipped with a Dean–Stark attachment for receiving the n-butanol-water or 2-ethylhexanol-water azeotrope and returning the n-butanol or 2-ethylhexanol to the reaction medium.

Resin modified with n-butanol:

FT-IR (film): 3400 cm^−^^1^ (ν, O–H groups); 3075, 1600, 1500 cm^−^^1^ (ɣ, =C–H and C=C in aromatics); 2920, 2877 cm^−^^1^ (ɣ, C–H in –CH_2_ groups); 1484 cm^−^^1^ (δ, C–H in –CH_2_ groups); 1209 cm^−^^1^ (ɣ C–O and δO–H in alcohols and phenols); 1115–879 cm^−^^1^ (δ in plane bending, C–H in substituted aromatics), 800–700 cm^−^^1^ (δ out of plane bending, C–H in substituted aromatics).

^1^H NMR (300 MHz, (CD_3_)_2_CO, δ): 7.18–7.56 (C_10_–H); 6.40–7.14 (C_3_–H; C_5_–H; C_16_–H; C_18_–H); 5.96–6.40 (C_11_–H; C_12_–H); 5.42–5.96 (C_11_-H; C_12_-H); 4.14–4.56 (C_20’_–Ha,b,K–CH_2_–O–CH_2_–K); 3.73–3.96 (F–CH_2_–O–, F–CH_2_–O–H); 3.45–3.63 (–CH_2_–O–, a); 2.73–3.28 (H_2_O); 1.73–2.47 (C_7_–H_3_, C_19_–H_3_, C_8_–H, (CD_3_)_2_CO); 1.19–1.68 (–CH_2_–, b, c); 0.72–1.00 (–CH_3_–, d).

Resin modified with 2-ethylhexanol:

FT-IR (film): 3400 cm^−^^1^ (ν, O–H groups); 3075, 1600, 1500 cm^−^^1^ (ɣ, =C–H and C=C in aromatics); 2920, 2877 cm^−^^1^ (ɣ, C–H in –CH_2_ groups); 1484 cm^−^^1^ (δ, C–H in –CH_2_ groups); 1209 cm^−^^1^ (ɣ C–O and δO–H in alcohols and phenols); 1035cm^−^^1^ (ν, –C–O–C– in ether groups); 1115–879 cm^−^^1^ (δ in plane bending, C–H in substituted aromatics), 800–700 cm^−^^1^ (δ out of plane bending, C–H in substituted aromatics).

^1^H NMR (300 MHz, (CD_3_)_2_CO, δ): 7.60–7.18 (C_10_–H); 7.18–6.41 (C_3_–H; C_5_–H; C_16_–H; C_18_–H); 6.39–5.97 (C_11_–H; C_12_–H); 5.95–5.46 (C_11_–H; C_12_–H); 5.32–4.89; 4.60–4.15 (C_20’_–Ha,b, K–CH_2_–O–CH_2_–K); 4.07–3.58 (F–CH_2_–O–, F–CH_2_–O–H); 3.58–3.33 (–CH_2_–O–, e); 3.20–2.72 (H_2_O); 2.49–1.66 (C_7_–H_3_, C_19_–H_3_, C_8_–H, (CD_3_)_2_CO); 1.60–1.02 (–CH–, –CH_2_–, f, g, i, j, k); 1.02–0.58 (–CH_3_–, l).

#### 2.2.4. Coating Preparation

The modified resins obtained immediately after the synthesis were applied to acid-resistant steel plates with a size of 15 cm × 7 cm (grade 1.4301, symbol EN X5CrNi18-10), creating a layer with a thickness (wet coating) of 30 µm. The coatings were dried at room temperature (solvent evaporation within 24 h) and thermally at 150 °C within 1 hr. The resultant coatings were subjected to basic tests to determine their physico-mechanical properties.

#### 2.2.5. Analytical Methods

##### High-Performance Liquid Chromatography (HPLC)

The content of unreacted compounds o–cresol and furfural was determined by high-performance liquid chromatography (HPLC), using Agilent Technologies 1260 Infinity II chromatograph with non-polar packed columns (reversed-phase). The resin test sample was dissolved in water–methanol mixture and separated on non-polar packed columns.

##### Analysis of Free Formaldehyde

The free formaldehyde content was determined using the hydroxylamine hydrochloride method, according to ISO 9397 [27]. The free formaldehyde present in the analytical sample was converted to oxime under the action of hydroxylamine hydrochloride. The hydrochloric acid formed during the reaction was titrated potentiometrically with a sodium hydroxide solution.

##### Analysis of Non-Volatile Substances

The content of non-volatile substances was determined according to ISO 8618. The non-volatile content was determined as the mass percentage of residue remaining after evaporation of the volatile components. The temperature of 135 °C, along with time and other conditions as per ISO 8618, were used [28].

##### Viscosity Determination

The viscosity of the resin was determined at 20 °C using a Brookfield viscometer. A spindle number 31 rotating at 160–200 rpm was used to measure viscosity, in accordance with PN-86/C-89085/06 [29].

##### Electrospray Ionization Mass Spectrometry (ESI-MS)

Using ESI-MS method, mass spectra of unmodified and etherified resins were recorded, along with the molecular masses. ESI-MS spectra were recorded on 4000Q TRAP spectrometer (AB SCIEX). Approximately 20 µg of the sample was dissolved in 10 mL of methanol. For ESI-MS analysis, the sample was diluted with 10× methanol solution. Samples with visible precipitate were filtered before analysis.

##### Nuclear Magnetic Resonance (NMR) Spectroscopy

^1^H NMR, ^13^C NMR and heteronuclear single quantum correlation (HSQC) spectra of unmodified resin were recorded on a Varian 600 MHz spectrometer using acetone-d6 as the solvent and TMS as an internal standard. ^1^H NMR spectra of resins modified with n-butanol and 2-ethylhexanol were recorded on a Unity INOVA 300 MHz (Agilent Technologies, Santa Clara, CA, USA) using acetone-d6 as the solvent and TMS as an internal standard.

##### Fourier Transform Infrared Spectroscopy (FT-IR)

Using a Nicolet 6700 FT-IR spectrometer Thermo Fisher (Waltham, MA, USA) with OMNIC™ Series software from based on PN-ISO 6286 [30], spectra were recorded in the bandwidth range from 4000 to 450 cm^−^^1^. The unmodified resin sample was mixed with KBr and made into a lozenge. The samples of modified resins were directly applied to an NaCl crystal. Each spectrum represents an average of 16 scans with a resolution of 4 cm^−^^1^.

##### Differential Scanning Calorimetry (DSC)

Using differential scanning calorimetry (Mettler Toledo apparatus), thermal properties were determined for unmodified and modified resin, in the temperature range from −40 to 240 °C at a heating rate of 10 K/min.

##### Gel Permeation Chromatography (GPC)

The GPC analysis was performed using a gel permeation chromatograph equipped with Viscotek VE3580 refractometer detector, pump L-7100 (Merck Hitachi, Tokyo, Japan) with Knauer degasser and 20 µL manual dosing valve. And equipped with series connected pre-column and 2 PlgelMiniMIX E 3 µm × 250 mm × 4.6 mm columns (Polymer Laboratories, Lewiston, ME, USA) operating at ambient temperature, tetrahydrofuran (THF) eluent flow rate of 0.3 mL/min. Approximately 50 mg of the test sample was weighed out in a 10 mL volumetric flask and filled up to the mark with THF. The solutions were subjected to chromatographic analysis under constant conditions.

## 3. Results and Discussion

### 3.1. Synthesis of o–Cresol–Furfural Resin

**Figure 1:**
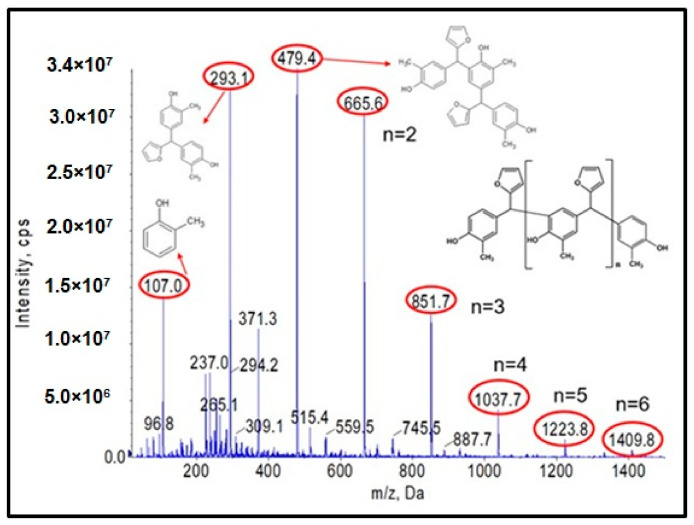
Negative ion mode ESI-MS mass spectrum for o–cresol–furfural resin at a molar ratio of 1:1.3, in the presence of 0.21 wt.% Na_2_CO_3_ at 120–150 °C after a condensation reaction of 34 h.

As a first step, studies were carried out to obtain a resole-type o–cresol–furfural resin with free hydroxymethylfurfural groups needed for modification by etherification with alcohols. A range of conditions were used for the condensation reaction of the o–cresol and furfural in order to obtain the desired product. The reaction conditions were selected on the basis of the literature data on the reaction of phenol with furfural [4,13] and our own preliminary research performed on o–cresol and furfural. The reaction was carried out at a molar ratio of 1:1.1–2.0, respectively, in alkaline media Na_2_CO_3_, K_2_CO_3_, NaOH, or KOH, at a reaction temperature of 120 to 150 °C over a period of 5 to 80 h. A molar excess of furfural was used to obtain resole-type resins containing hydroxyl groups. After each synthesis, a sample of the resulting resin was subjected to complete physicochemical characterization (Table 1) and ESI-MS analysis (Figure 1).

Based on the available literature data, it was determined that the reaction proceeds according to the mechanism known for phenol–formaldehyde resins [31]. Detailed studies on the kinetics of phenol–furfural novolac resins, described by Ahuja et al. [13], showed that when reacted with phenol, furfural is much less reactive than formaldehyde. The reason for this is the occurrence of a spatial defect in the furfural molecule and therefore a longer condensation time is needed to achieve a higher degree of reactivity.

After the condensation reaction, an o–cresol–furfural resin was obtained with unreactedo–cresol in the range of 1.2 to 8.6 wt.%, and unreacted furfural in the range of 12.3–29.8 wt.%. The obtained resins varied in consistency from solid to liquid.

Figure 1 shows the ESI-MS spectrum of o–cresol–furfural resin obtained with a 1:1.3 molar ratio of o–cresol and furfural, in the presence of 0.21 wt.% of Na_2_CO_3_ at 120–150 °C after a condensation reaction of 34 h. Based on the molecular masses corresponding to the individual molecular ions, the likely structures of the oligomeric compounds contained in the resin were determined.

**Figure 2:**
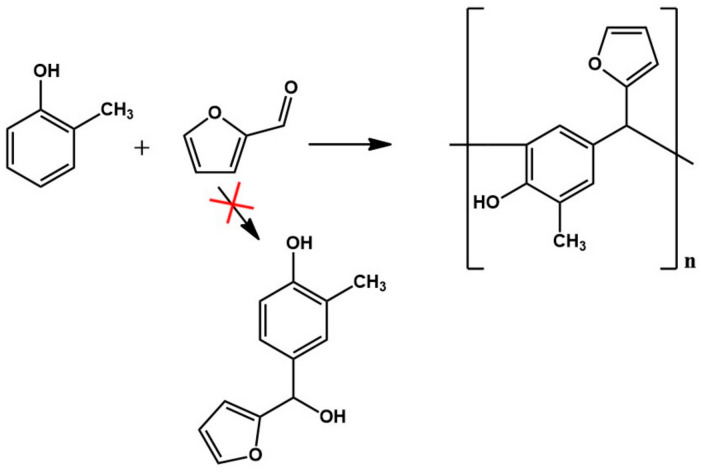
Reaction scheme of the condensation of o–cresol and furfural.

The analysis of the ESI-MS mass spectrometry showed that the resulting o–cresol–furfural resin contained oligomeric compounds with the structure shown in Figure 2, composed of 3 to 7 repeating mers. However, the ESI-MS spectra did not show the presence of peaks corresponding to structures containing hydroxymethylfurfural groups, which are necessary for the modification reaction with alcohols.

Despite attempting a wide range of conditions for the synthesis of an o–cresol–furfural resin (i.e., the reaction temperature, concentration, and type of catalyst, as well as the molar ratio of o–cresol to furfural), it was not possible to obtain a resin with hydroxymethylfurfural groups suitable for carrying out the modification with alcohols. Therefore, in the next stage of the study, the concept was changed, and it was decided to introduce hydroxyl groups into the o–cresol–furfural resin by using a limited amount of formaldehyde for the synthesis.

### 3.2. Synthesis of o–Cresol–Furfural–Formaldehyde Resin

**Figure 3:**
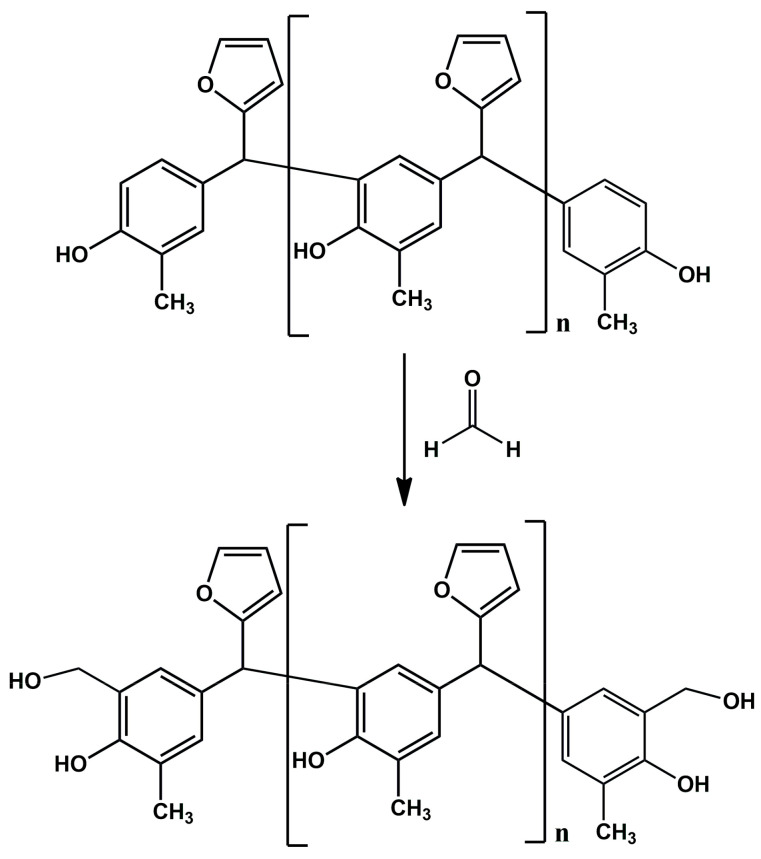
Reaction scheme of activation of o–cresol–furfural resin with hydroxymethylene groups.

In the reaction of o–cresol with furfural, carried out in an alkaline medium, it was not possible to obtain a resin containing the hydroxymethylfurfural groups needed for the modification with alcohols. Therefore, in subsequent syntheses formaldehyde was introduced into the reaction system in order to activate the o–cresol–furfural resin with hydroxymethylene groups (Figure 3). The formaldehyde was introduced after the completion of the o–cresol–furfural condensation reaction.

**Figure 4:**
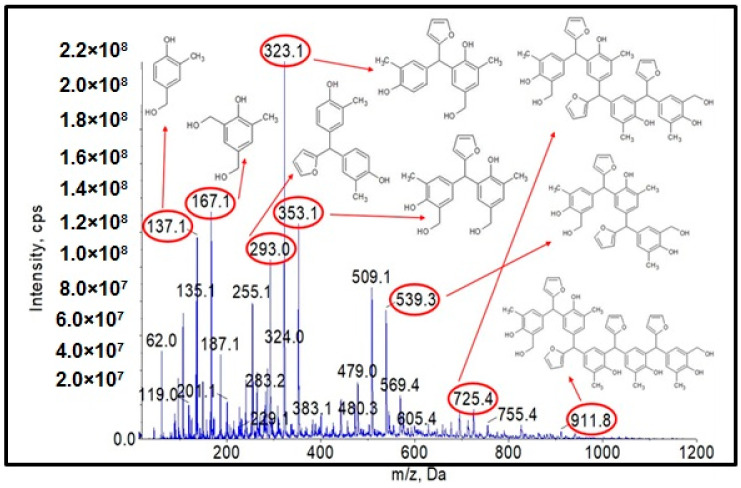
ESI-MS mass spectrum in negative ion mode for the o–cresol–furfural–formaldehyde resin.

The o–cresol–furfural condensation reaction was carried out in an alkaline medium (10% KOH solution), with an o–cresol to furfural molar ratio of 1:0.5 to 1:0.8 at 120 °C for 11 h. After this time, formaldehyde in the form of a 46% formalin solution at a molar ratio of 0.5–0.8 mole/1 mole o–cresol was added to the reaction system. The reaction was carried out at 90 °C for 2 h. After completion of the synthesis, a sample of the resulting resin was subjected to full physicochemical characterization (Table 2) and ESI-MS analysis (Figure 4) to check whether structures containing hydroxymethylene groups were present in the resulting resin. Selected resins were also subjected to FT-IR and ^1^H NMR analysis. Thermal properties of selected resins were determined using DSC.

After a series of syntheses, the process conditions under which an unmodified o–cresol–furfural–formaldehyde resin, containing free hydroxymethylene groups in which the most favorable physicochemical properties is obtained, were selected. The characteristics of the chosen resin and the process parameters under which they were obtained are shown in Table 3. In the first stage of the o–cresol–furfural condensation reaction, a molar ratio of 1:0.65 was chosen and the reaction was carried out at 120 °C for 11 h; under these conditions, furfural was reacted to a content of 0.2%. In the second condensation step, 0.65 mole of formaldehyde (46% formalin solution) was added and the reaction proceeded at 90 °C for 1 h. The final concentration of o–cresol in the resin was measured at 2.2%.

Figure 4 shows the obtained ESI-MS spectrum for the unmodified o–cresol–furfural–formaldehyde resin selected for further study due to its physicochemical parameters (Table 3). The ESI-MS spectrum shows molecular peaks corresponding to the molecular weights of the oligomeric compounds present in the resin. The probable structures of the compounds present in the obtained resin are also shown in Figure 4. A large number of structures contain hydroxyl groups. This was promising with regards to further research into the modification of such resins.

**Figure 5:**
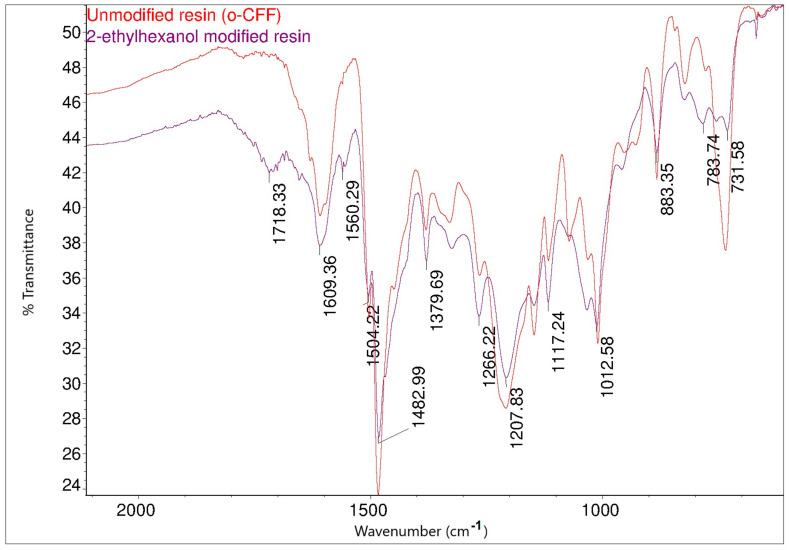
FT-IR spectrum of unmodified o–cresol–furfural–formaldehyde resin.

The presence of hydroxyl groups in the obtained resin was also confirmed by analysis of FT-IR and ^1^H NMR spectra. The obtained FT-IR spectrum is shown in Figure 5. In the FT-IR spectrum, the bands at 3400 cm^−1^ (O–H stretching vibrations in alcohol or acids), were clearly visible in addition to other distinctive bands.

**Figure 6:**
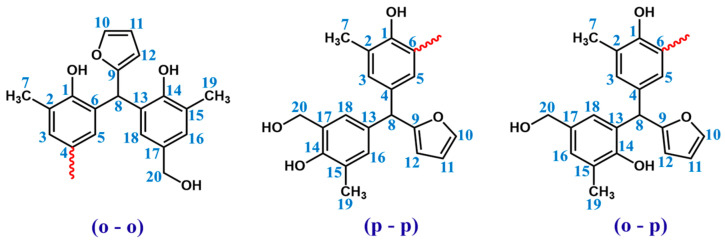
Possible structures along with the numbered carbon atoms (red wavy lines indicate possible attachment sites for subsequent molecules).

In order to determine the structure of resin ^1^H and ^13^C NMR, spectra were recorded. The signals of protons and carbons assigned based on one-dimensional spectra are presented in Table 4. Chemical structure of the o–cresol–furfural–formaldehyde resin was confirmed, considering three possible structures, presented in Figure 6.

**Figure 7:**
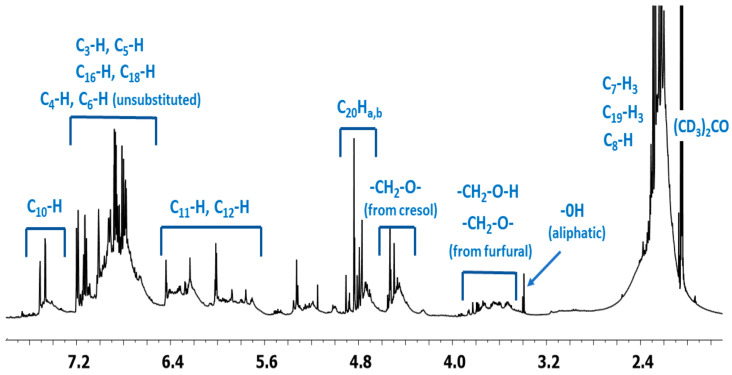
^1^H NMR spectrum of o–cresol–furfural–formaldehyde resin.

^1^H NMR spectrum of the o–cresol–furfural–formaldehyde resin (Figure 7) showed a peak in the range of 6.50 to 7.21 ppm characteristic for the aromatic cresol ring protons; protons in meta position (C_3_–H; C_5_–H; C_16_–H; C_18_–H) and unsubstituted ortho (C_6_–H) and para (C_4_–H) position. The signals at δ = 5.68–6.45 ppm (C_11_–H; C_12_–H) and δ = 7.25–7.50 (C_10_–H) ppm which could be assigned to protons in furfural ring are also visible. The presence of signals in the range of4.83–4.68 ppm (C_20_–Ha,b) and 4.52–4.37 ppm (K–CH_2_–O–CH_2_–K) confirms the addition of formaldehyde to the o–cresol and the formation of hydroxymethylene groups, as well as their partial condensation with the formation of dimethylene ether bridges. The presence of such structures has also been identified on the base of ESI-MS spectrum (Figure 4).

**Figure 8:**
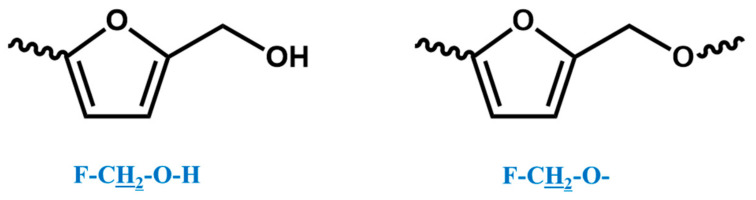
Structures of furfurals with –CH_2_–OH and –CH_2_–O– groups attached.

In the ^1^H NMR spectrum of the o–cresol–furfural–formaldehyde resin, additional signals in the range of 3.70–3.57 ppm were also found, and assigned to methylene protons of –CH_2_–OH and –CH_2_–O– groups attached to the furfural (Figure 8). Chemical structures, such as in Figure 8, were not identified on the basis of ESI–MS spectrum (Figure 4) due to the molecular peaks being indiscriminate of the CH_2_OH groups attachment to the cresol or furfural.

In order to definitively verify the structure of the o–cresol–furfural–formaldehyde resin, a two-dimensional HSQC spectrum was also performed, allowing proton signals to be assigned to the corresponding carbon atoms and all signals to be described in the ^1^H and ^13^C NMR spectra.

**Figure 9:**
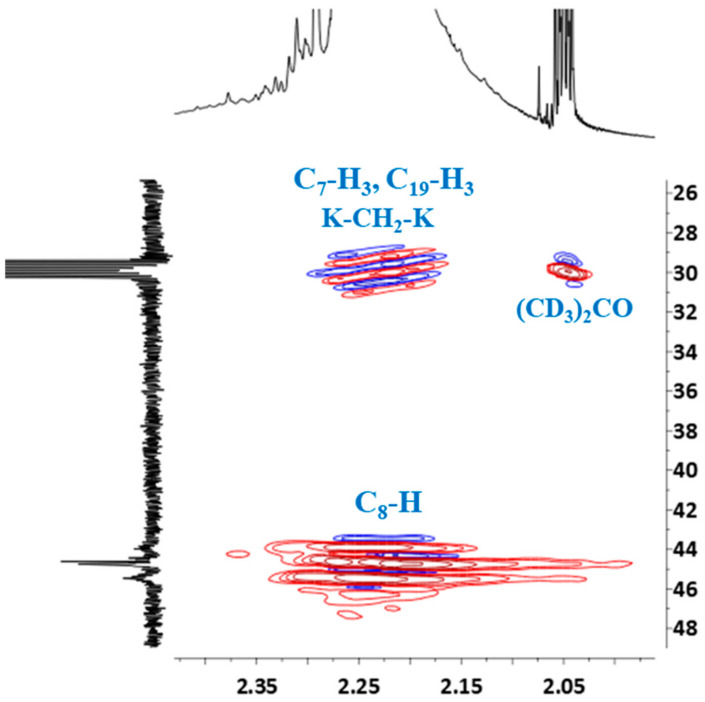
Partial HSQC spectrum showing the signals of methyl groups and protons of –CH– linkage (C_8_–H): ortho-ortho (o-o), ortho-para (o-p) and para-para (p-p).

HSQC spectrum confirmed the presence of the signal of methyl groups in ^13^C NMR spectrum, which overlapped with the signal of deuterated acetone (Figure 9). The signal of (CD_3_)_2_CO also overlapped the signal of the methylene bridge between the two cresols (K–CH_2_–K).

**Figure 10:**
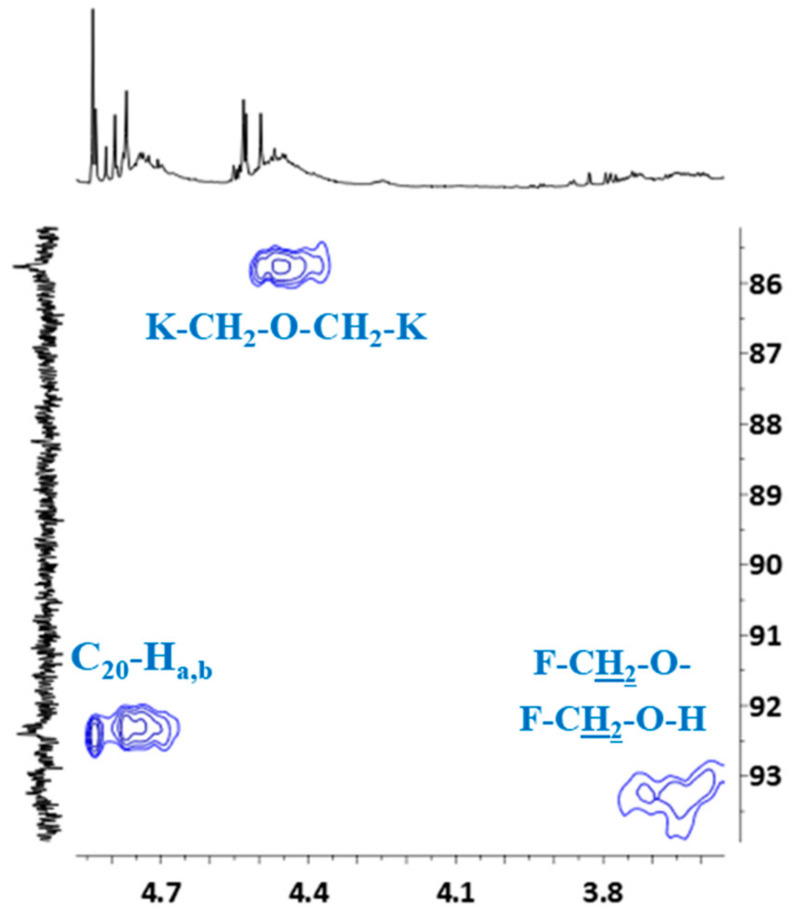
Partial HSQC spectrum showing the signals of hydroxymethylene groups, dimethylene ether bridges, protons and signals of –CH_2_–OH and –CH_2_–O– groups attached to furfural.

HSQC spectrum also confirmed the previous signal assignment of hydroxymethylene groups (C_20_–Ha,b) and dimethylene ether bridges (K–CH_2_–O–CH_2_–K), as well as the presence of signals –CH_2_–OH and –CH_2_–O– groups attached to the furfural (Figure 10).

ESI-MS, FT-IR, and NMR spectra confirm the presumed structure of the o–cresol–furfural–formaldehyde resin, as well as the presence of hydroxymethylene groups in the resin. This indicated the possibility of modifying such resin with selected alcohols in the etherification process. The unmodified o–cresol–furfural–formaldehyde resin had a molecular weight of 1245 g/mol, non-volatile content of 98%, and was in a solid state.

**Figure 11:**
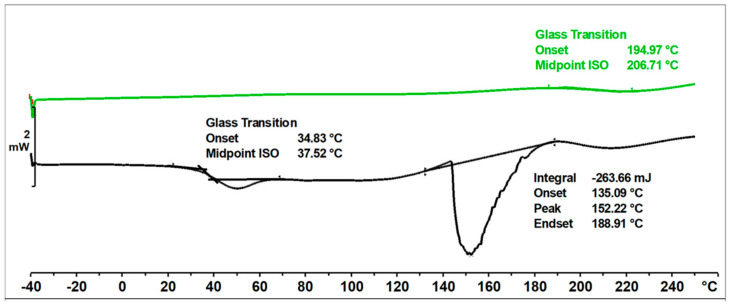
DSC thermogram of unmodified o–cresol–furfural–formaldehyde resin.

In the DSC thermogram (Figure 11) of the unmodified o–cresol–furfural–formaldehyde resin, in the first heating run, a glass transition temperature of T_g_ = 37.5 °C was observed, as well as an endothermic transformation with a peak temperature of T = 152 °C and a transition heat of ∆H = −264 J/g. This transformation was related to the melting of the resin, but probably also due to the condensation reaction of the hydroxymethylene groups and the evaporation of the condensation water. Heating the resin under DSC test conditions lead to crosslinking of the resin. After heating to 250 °C, the resin became non-melting and in the second heating run, the transformation indicating melting or further condensation of the resin was not observed. This means that the thermogram recorded in the second run was the thermogram for the cured resin. The glass transition temperature of the unmodified resin in the second heating run was very high at T_g_ = 207 °C, indicating good thermal resistance and high stiffness over a wide temperature range of the cured resin.

### 3.3. Etherification of o–Cresol–Furfural–Formaldehyde Resin with n-Butanol

The next step in the ongoing research was to carry out the etherification reaction of the selected o–cresol–furfural–formaldehyde resin (Figure 3) with n-butyl alcohol applied at 40 wt.%, 60 wt.%, or 80 wt.%. The etherification reaction was conducted in the presence of a range of dicarboxylic acids used as a catalyst: oxalic, malonic, adipic, succinic, or sebacic ones. The amounts of acids ranged from 0.002 to 0.15 mole/per mole of o–cresol, for the appropriate amount of n-butanol to give the corresponding pH = 6–5, pH = 5–4, or pH = 4–3.

The n-butyl alcohol and the appropriate dicarboxylic acid were added to the unmodified o–cresol–furfural–formaldehyde resin, and the reaction system was then heated to 110 °C by azeotropic distillation until the water was completely removed from the system, with an approximate reaction time of 10–20 h. After completion of the reaction, unreacted n-butanol was evaporated from the resin and the resulting etherified resin was subjected to ESI-MS analysis.

**Figure 12:**
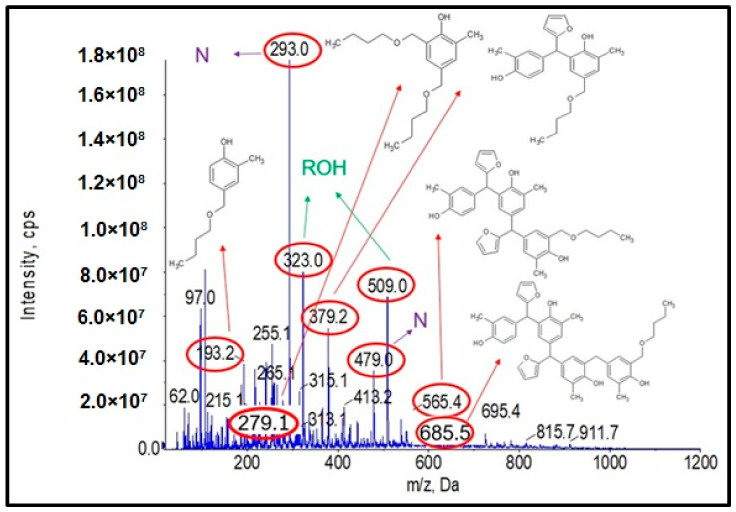
Mass spectrum of ESI-MS in negative ion mode for the o–cresol–furfural–formaldehyde resin etherified with n-butanol, in the presence of malonic acid used as a catalyst.

**Figure 13:**
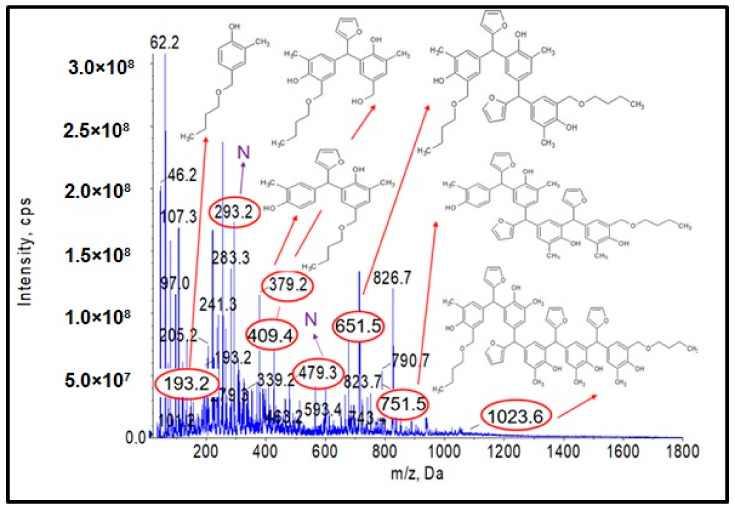
Mass spectrum of ESI-MS in negative ion mode for o–cresol–furfural–formaldehyde resin etherified with n-butanol in the presence of oxalic acid used as a catalyst.

Analysis of the ESI-MS mass spectra confirmed the assumed course of the reaction. In the obtained ESI-MS spectra for the n-butanol modified o–cresol–furfural–formaldehyde resin, molecular peaks corresponding to resin molecules with attached oxybutyl groups were observed (Figure 12 and Figure 13). On the basis of the ESI-MS spectra obtained, it was concluded that n-butanol etherification occurs to the greatest extent with 80 wt.% n-butanol in an oxalic or malonic acid medium. The parameters of such reactions are summarized in Table 5, and the physicochemical properties of the modified resins in Table 6.

After an etherification reaction, with an excess of n-butanol of 80% wt. per resin, the modified o–cresol–furfural–formaldehyde resins were a liquid with a viscosity of 22.5 mPa·s when malonic acid was used as a catalyst, or 12.1 mPa·s when oxalic acid was used as a catalyst. Modified resins had a molecular weight of 3402 g/mol or 11,389 g/mol and a non-volatile content of 17.52 or 19.91%, respectively. The resins’ samples were dried at room temperature before determining the molecular weight and thermal properties. After drying at room temperature, the resins were solid and their glass transition temperatures were 55 and 53 °C, respectively.

A series of molecular peaks visible in the ESI-MS spectra (Figure 12 and Figure 13) correspond to the o–cresol–furfural–formaldehyde resin molecules containing butyl groups in their structure, linked to the resin molecule by an ether bond. Figure 12 and Figure 13 show also selected structures assigned to the corresponding molecular peaks.

On the mass spectra (Figure 12 and Figure 13), in addition to molecular peaks corresponding to etherified molecules, peaks assigned to molecules that have not etherified and still contain hydroxymethylene groups (marked on the ESI-MS spectra, with the symbol ROH) were also observed. Some peaks (marked on the ESI-MS spectra, with the symbol N) were indicative of the presence of molecules that did not acquire hydroxymethylene groups in the previous steps and therefore could not be etherified. However, the proportion of molecular peaks corresponding to unmodified and unreacted molecules is small, and the presence of molecules containing free hydroxymethylene groups should promote the curing process of the etherified resin.

Samples of the butanol-modified resin, under the conditions described in Table 5, were also subjected to FT-IR and ^1^H NMR spectroscopic studies, and their thermal properties were also investigated by DSC.

**Figure 14:**
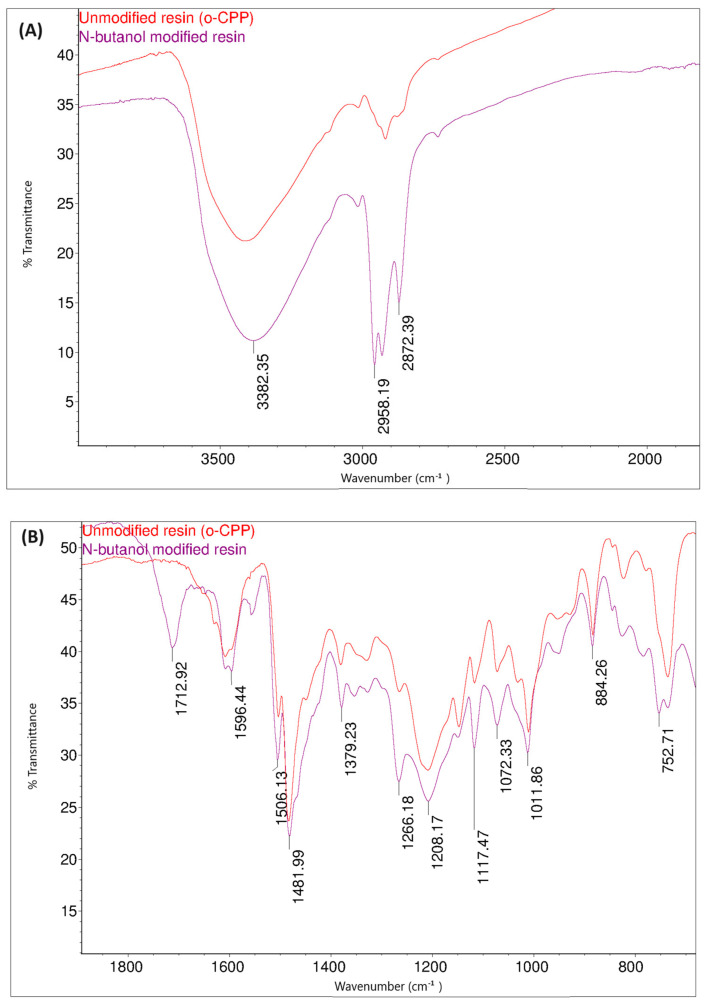
FT−IR spectra of o–cresol–furfural–formaldehyde resin, unmodified and after modification with n-butanol: (**A**) range 4000–2500 cm^−^^1^; (**B**) range 1700–600 cm^−^^1^.

Figure 14 shows the FT-IR spectra of the o–cresol–furfural–formaldehyde resin before and after the modification with butanol. In the FT-IR spectrum of the modified resin, the band arrangement characteristic of n-butyl groups (bands at 2960, 2932, 2870 cm^−^^1^ from C–H stretching vibrations in CH_3_ and CH_2_ groups) are visible, in addition to other bands also identified for the unmodified resin. In the FT-IR spectra of the modified resin, the presence of bands corresponding to the C–O–C bond vibrations could not be unambiguously confirmed. However, because the bands associated with the presence of ether groups (1000–1035 cm^−^^1^) are bands of low intensity, and because their detection range overlaps with many other bands corresponding to C–C bond stretching vibrations, the invisibility of these signals does not prejudge the absence of ether groups in the molecules. In the FT-IR spectra of the modification products, additional bands are also visible at 1713 cm^−1^, originating from stretching vibrations of C=O, carboxylic acids, and at 1558 cm^−1^, originating from COO– groups, which may originate from salts of carboxylic acids used as catalysts.

**Figure 15:**
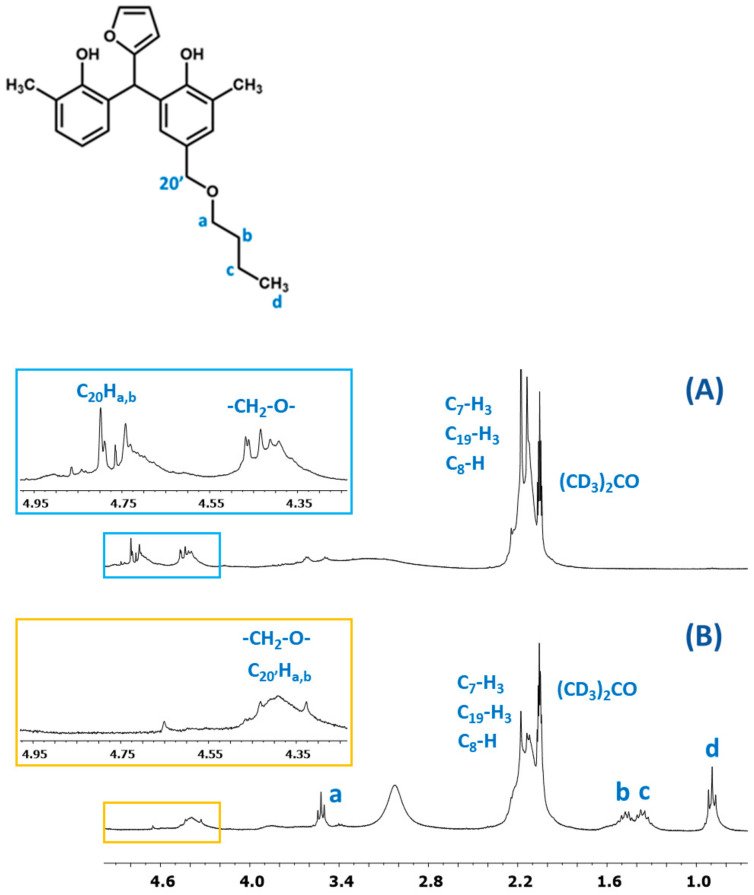
The ^1^H NMR spectrum of o–cresol–furfural–formaldehyderesin: (**A**) unmodified and (**B**) modified with n-butanol.

The presence of butyl groups in the modified resin was also confirmed by ^1^H NMR analysis. Figure 15 shows the fragments of the^1^H NMR spectra of the o–cresol–furfural–formaldehyde resins before and after etherification. The^1^H NMR spectrum of the modified resin (Figure 15B) showed signals corresponding to the protons of the butyl groups (d, c, b, and a). The presence of these signals in the ^1^H NMR spectrum with the simultaneous disappearance of the signal of methylene protons of the -CH_2_OH groups confirms the etherification of the resins with butanol.

The ESI-MS, FT-IR, and NMR spectra confirmed the occurrence of an etherification reaction between the unmodified resole resin and n-butyl alcohol and thus a successful preparation of a resin with oxybutyl groups attached to the oligomeric chains of the o–cresol–furfural resin. The etherified resin has a lower viscosity and is more flexible compared to the resin before modification. The glass transition temperature of the etherified resin recorded in the second heating run is T_g_ = 164 °C, which is lower than the glass transition temperature determined for the unmodified o–cresol–furfural–formaldehyde resin (T_g_ = 207 °C).

**Figure 16:**
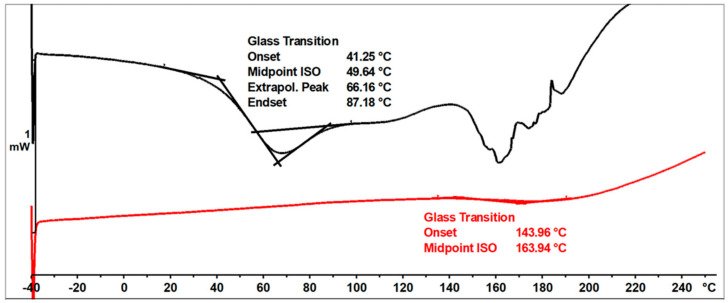
DSC thermograms of o–cresol–furfural–formaldehyde resin modified with n−butanol.

Figure 16 shows DSC thermograms of the n-butanol etherified resin recorded in the first and second run of heating.

The glass transition temperature of the n-butanol etherified resin, determined by DSC in the first run of heating was T_g_ = 50 °C. An endothermic transformation was also observed in the first heating run, starting at a temperature above 140 °C. This transformation was likely related to the condensation reaction and the evaporation of its by-products. As a result of the condensation, the resin cured. The thermogram recorded in the second run characterized the cured resin and therefore no transformation was observed in the range of temperatures studied, except for the glass transition temperature, which was higher than in the first run at T_g_ = 164 °C.

### 3.4. Etherification of o–Cresol–Furfural–Formaldehyde Resin with 2-Ethylhexanol

**Figure 17:**
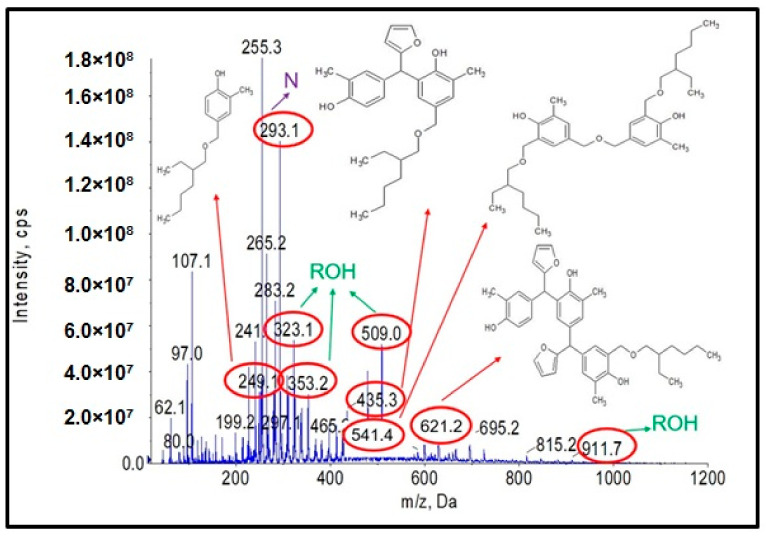
ESI-MS mass spectrum in negative ion mode for resin o–cresol–furfural–formaldehyde resin after etherification reaction with 2-ethylhexanol in the presence of malonic acid used as a catalyst.

**Figure 18:**
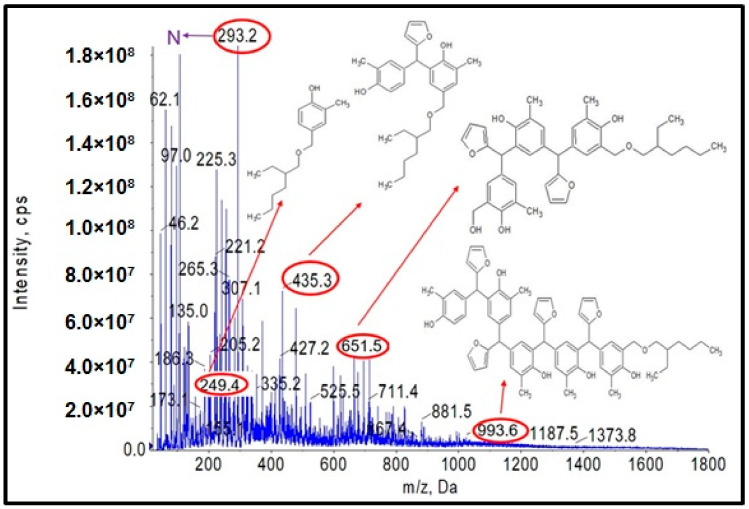
ESI-MS mass spectrum in negative ion mode for resin o–cresol–furfural–formaldehyde resin after etherification reaction with 2-ethylhexanol in the presence of oxalic acid used as a catalyst.

Another objective of the planned studies was to test the possibility of obtaining an o–cresol–furfural–formaldehyde resin etherified with 2-ethylhexanol. The etherification with 2-ethylhexanol was carried out analogously to that with n-butanol, using 40 wt.%, 60 wt.%, or 80 wt.% alcohol per resin, in the presence of the dicarboxylic acids. The resin containing hydroxymetyhylene groups (Figure 4), with the properties described in Table 3, was etherified. To the unmodified resin was added an excess of 2-ethylhexyl alcohol and an appropriate amount of catalyst (0.002–0.15 moles of malonic acid or oxalic acid for 1 mole of o–cresol, for an appropriate amount of 2-ethylhexanol to give appropriate pH = 6–5, pH = 5–4, or pH = 4–2.5). The reaction system was heated to 110 °C and the reaction was carried out using azeotropic distillation until water was completely removed from the system after approximately 10–20 h. After completion of the reaction, unreacted 2-ethylhexanol was evaporated from the resin and the reaction product was subjected to ESI-MS analysis. Analysis of the ESI-MS mass spectra confirmed the assumed course of the reaction. In the ESI-MS spectra obtained for the o–cresol–furfural–formaldehyde resin modified with 2-ethylhexanol, molecular peaks corresponding to resin molecules that had undergone 2-ethylhexanol etherification were observed (Figure 17 and Figure 18). On the basis of the obtained ESI-MS spectra, 2-ethylhexanol etherification was found to occur to the greatest extent with 80 wt.% alcohol in an oxalic or malonic acid media. The parameters of such reactions with the highest efficiency are summarized in Table 7 and the physicochemical properties of the modified resins in Table 8.

After the etherification reaction, with an excess of 2-ethylhexanol in the amount of 80 wt.% per resin, the modified o–cresol–furfural–formaldehyde resins were liquid with a viscosity of 248 or 12.1 mPa·s when malonic or oxalic acid was used as a catalyst, respectively. The modified resins had a molecular weight of 6394 or 8334 g/mol and a non-volatile content of 20.57 or 19.91%, respectively. Before determining the molecular weight, the resins samples were dried at room temperature. After drying, the modified resins were solids with a glass transition temperature of 48 and 53 °C, respectively.

In Figure 17 and Figure 18, the probable structures of oligomeric molecules containing 2-etylohexyl groups attached by ether bond, whose molecular weights correspond to the molecular peaks observed in the ESI-MS mass spectra, were shown.

In the mass spectra of the o–cresol–furfural–formaldehyde resin etherified with 2-ethylhexanol (Figure 17 and Figure 18), molecular peaks corresponding to molecules that do not contain the 2-ethylhexyl group were also present. Some of them were unreactive molecules, which did not have groups capable of etherification (the peaks corresponding to such molecules are marked on ESI-MS spectra with the symbol N), while some were molecules having unreacted hydroxymethylene groups, which did not undergo etherification (the peaks corresponding to such molecules are marked on ESI-MS spectra with the symbol ROH). The presence of unreacted hydroxymethylene groups in the modified resin may contribute to the curing process of the etherified resin in a condensation reaction, which can be advantageous.

**Figure 19:**
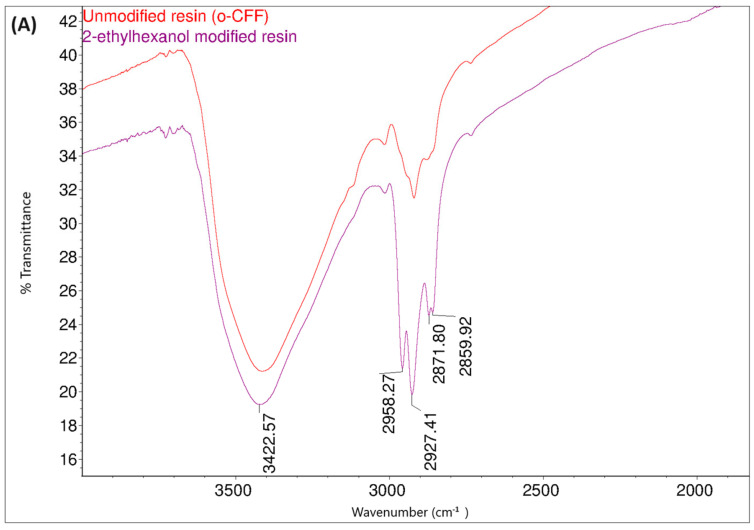

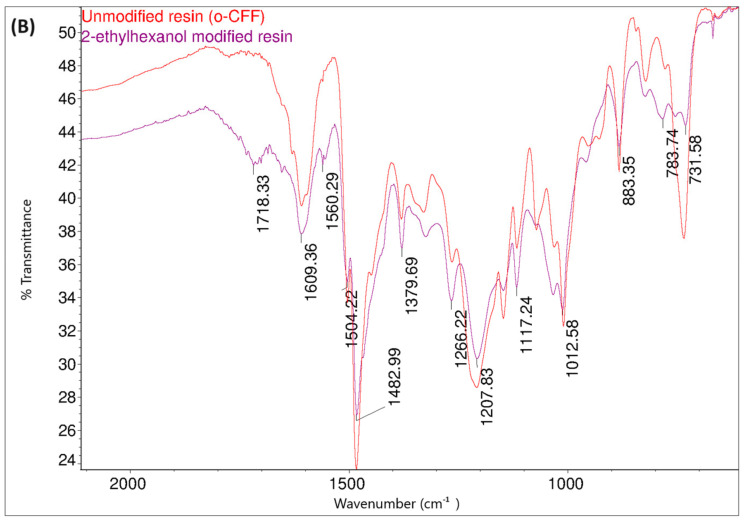
FT-IR spectraof o–cresol–furfural–formaldehyde resin unmodified and modified with 2-ethylhexanol: (**A**) range 4000–2500 cm^−^^1^; (**B**) range 1700–600 cm^−^^1^.

The structure of the resin modified with 2-ethylhexanol was also confirmed by FT-IR and ^1^H NMR spectroscopic methods. Figure 19 shows the FT-IR spectra of the o–cresol–furfural–formaldehyde resin before and after the modification with 2-ethylhexanol.

Comparing the FT-IR spectra of the resin before and after the 2-ethylhexanol etherification, it can be seen that in the spectrum of the modification resin, in addition to the bands characteristic of the initial o–cresol–furfural–formaldehyde resin, clearly visible bands of C-H stretching vibrations in the CH_3_ and CH_2_ groups (an arrangement of bands characteristic of 2-ethylhexyl group) are present at wavelengths: 2958, 2927, 2875, and 2860 cm^−^^1^. An additional band was also observed at 1035 cm^−^^1^ in the range of the C-O-C ether bond. The presence of this band unequivocally confirms the occurrence of the reaction between 2-ethylhexanol with hydroxymethylene groups.

**Figure 20:**
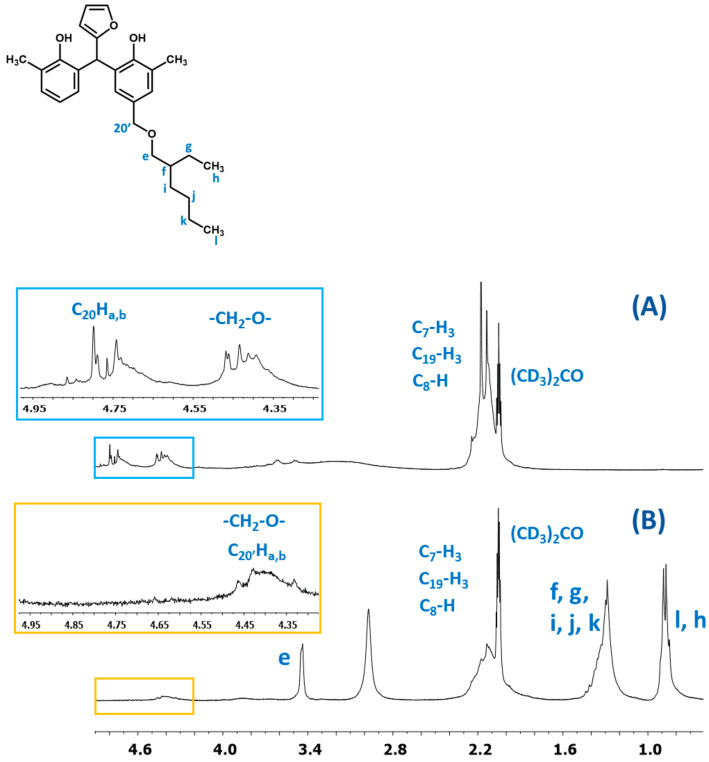
The^1^H NMR spectra of resin: (**A**) unmodified and (**B**) modified with 2-ethylhexanol.

In Figure 20, the fragments of the^1^H NMR spectra of the o–cresol–furfural–formaldehyde resins before and after etherification with 2-ethylhexanol are presented. The presence of signals assigned to 2-ethylhexanol (l, k, j, i, f, g, h, and e) and the disappearance of hydroxymethylene groups (C_20_Ha,b) indicate that a modified resin has been obtained.

ESI-MS, FT-IR, and NMR spectra confirm the occurrence of an etherification reaction between the unmodified resole resin and 2-ethylhexanol and thus a successful synthesis of a resin containing 2-ethylhexyl groups attached to oligomeric o–cresol–furfural–formaldehyde resin chains in its structure.

The o–cresol–furfural–formaldehyde resin etherified with 2-ethylhexanol had a lower viscosity and was more flexible than the resin before modification. The glass transition temperature of the 2-ethylhexanol-etherified resin recorded in the second heating run was T_g_ = 89 °C, which was lower than the glass transition temperature determined for both the unmodified resin (T_g_ = 207 °C), and the n-butanol etherified resin (T_g_ = 164 °C).

**Figure 21:**
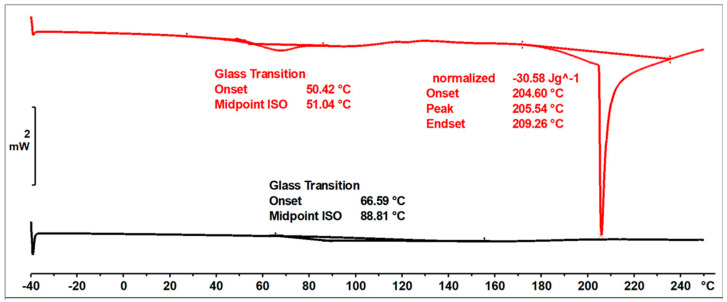
DSC thermograms of o–cresol–furfural–formaldehyde resin modified with 2−ethylhexanol in 1st and 2nd runs of heating.

Figure 21 shows the DSC thermograms of the 2-ethylhexanol modified resin, captured during two runs of heating, in the temperature range of −40 to 260 °C.

The glass transition temperature of the resin after etherification with 2-ethylhexanol, determined by DSC in the first heating run was T_g_ = 51 °C. In the first run of heating, an endothermic transformation was also observed, likely related to the condensation reaction and the curing process of the resin. This transformation was observed at 205 °C. In the second run, such a transformation was not observed, nor was any transformation related to the melting of the resin. The glass transition temperature recorded in the second run was higher, due to the crosslinking of the resin.

### 3.5. Application-Related Properties

Resins obtained by the etherification process were liquid, the viscosity at 20 °C was 12–248 mPa·s, and were soluble in non-polar solvents (xylene, toluene) with good overall stability. After one year of storage under laboratory conditions, they did not change their physicochemical properties and were still suitable for application. At 150 °C within 1 h, they cure, making them insoluble and non-fusible. The resulting resins had a free formaldehyde content of <0.1%.

**Figure 22:**
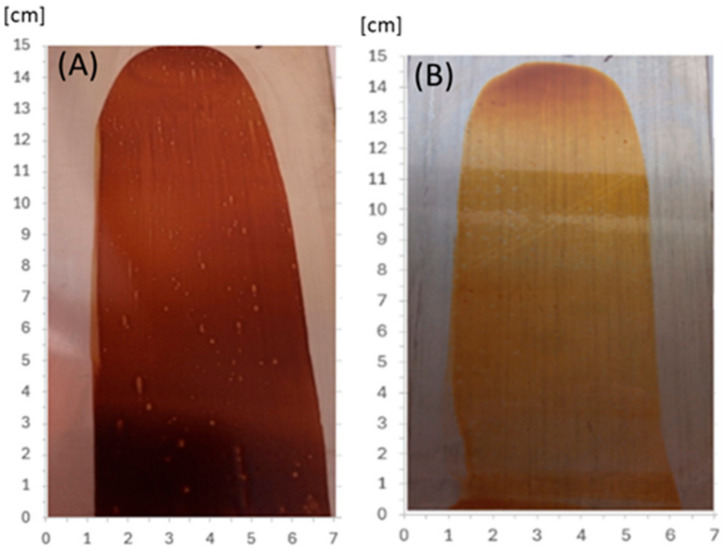
The coatings obtained from o–cresol–furfural–formaldehyde resins, modified with (**A**) n-butanol, (**B**) 2-ethylhexanol, on an acid-resistant plate, after drying at 150 °C for 1 h.

In order to ascertain the potential viability of the etherified resins in the paint industry, an initial attempt was made at creating and thermally crosslinking coatings. The modified resins, obtained immediately after the synthesis, were applied to acid-resistant steel plates, obtaining a layer with a thickness (wet coating) of 30 µm. The coatings were dried at room temperature (solvent evaporation within 24 h) and thermally at 150 °C within 1 h. After drying at room temperature, the resultant coating was hard, non-sticky, and had a good adhesion to the substrate, but remained soluble in organic solvents and sensitive to temperature (it melted when heated above 120 °C). After drying at 150 °C, the coating became non-meltable and insoluble in organic solvents (polar and non-polar), which means that the resin was successfully cured under these conditions. Figure 22 shows coatings of o–cresol–furfural–formaldehyde resins modified with n-butanol (Figure 22A) and 2-ethylhexanol (Figure 22B), respectively, obtained on an acid-resistant steel plate and cured for 1 h at 150 °C.

The resulting coatings were subjected to basic tests, accordingly to international standards [32,33,34,35], to determine their physico-mechanical properties. The properties of the coatings obtained from the o–cresol–furfural–formaldehyde resin modified with n-butanol or 2-ethylhexanol are shown in Table 9.

Above all, the obtained coatings showed very good adhesion to the substrate, 0 or 1 degree on a 6-point full scale. It confirms the literature data that furan resin, due to their excellent adhesion, are highly suitable for making protective coatings [1]. Although some limited information about the use of phenol–furfural compositions for obtaining protective coatings can be found in the literature, there is a lack of data about their basic properties, such as elasticity or hardness. This is probably due to the fact that unmodified phenol–furfural coatings are hard and rather inflexible due to the rigid chemical structure associated with the presence of aromatic rings and thus are not favorable for such applications. The resultant coatings based on etherified o–cresol–furfural–formaldehyde resins show very good flexibility (Table 9), especially when 2-ethylheksanol was used for the modification. The flexibility of these coatings is much higher than that of phenol–formaldehyde coatings modified with butanol or lower glycols [25], and comparable to that of phenol–formaldehyde coatings modified with polyetherols [25] or urethane prepolymers [36]. Modification of phenol–formaldehyde resins with polyetherols or urethane prepolymers offers the possibility of increasing the flexibility of coatings, but also results in a significant reduction in their hardness [25,36]. In contrast, our coatings obtained from etherified o–cresol–furfural–formaldehyde resins are characterized by very good hardness (Table 9) and at the same time are sufficiently flexible. The excellent balance of flexibility and hardness can be attributed to the presence of flexible aliphatic chains and benzene and furan rings in the polymeric chains.

Although only an initial small-scale test, the properties of obtained coatings, combined with the safety and environmental advantages, are favorable enough to warrant further research, which we are currently conducting.

## 4. Conclusions

The use of a two-stage condensation reaction of o–cresol with furfural and formaldehyde allowed us to obtain a resole type resin containing free hydroxymethylene groups, which can be used for etherification. An unmodified resin had low contents of unreacted raw materials including o–cresol 1%, furfural 0.2%, and formaldehyde < 0.1%, and a non-volatile content of 98%. The obtained resin is based on furfural, which is a widely available biomass-based raw material with much lower toxicity than formaldehyde. Although the synthesis still required the use of some formaldehyde as a reagent, the obtained resins had a free formaldehyde content of <0.1%, i.e., they meet the requirements on the reduction of volatile organic compounds [37]. Furthermore, the resin did not contain phenol, the use of which is also increasingly prohibited in a wide range of products.

The unmodified resin was subjected to an etherification reaction with an excess of n-butanol or 2-ethylhexanol and malonic or oxalic acid used as the catalyst. Similar to an unmodified base resin, the etherified o–cresol–furfural–formaldehyde resins had a low content of free o–cresol < 1 wt.%, furfural < 0.1 wt.%., and formaldehyde < 0.1 wt.%, and they could feasibly be used as an environmentally friendly alternative to traditional phenol–formaldehyde resins in the paint industry. They demonstrate the ability to create a protective coating with good adherence to metal substrates and the excellent balance of flexibility and hardness.

Furthermore, the successful increase in elasticity through the process of etherification of the o–cresol–furfural resins points at the possibility of adapting such resins to many other fields of application, where currently available unmodified resins do not meet the requirements for use. The modified resins stored at room temperature after one year do not change their physicochemical properties, which means that they are reasonably resistant to aging, which is another property desirable for industrial use.

## Figures and Tables

**Table 1:** Range of physicochemical properties of o–cresol–furfural resins obtained in a one-step condensation reaction.

Properties	Parameters
Molar ratio o–cresol–furfural	1:1.1–2
Reaction temperature, °C	120–150
Reaction time, hour	5–80
Free o–cresol content, wt.%	1.2–8.6
Free furfural content, wt.%	12.3–29.8
Physical state	Solid–liquid

**Table 2:** Range of physicochemical properties of o–cresol–furfural–formaldehyde resins after two-step condensation with formaldehyde in the second step.

Physicochemical Properties	Parameters
Molar ratio o–cresol–furfural–formaldehyde	1:0.5–0.8:0.5–0.8
Reaction temperature, °C (1st stage/2nd stage)	120/90
Reaction time, hours (1st stage/2nd stage)	11/2
Free o–cresol content, wt.%	4–14
Free furfural content, wt.%	0–4
Free formaldehyde content, wt.%	1–3
Physical state	Solid

**Table 3:** Physicochemical properties of the unmodified o–cresol–furfural–formaldehyde resin chosen for further alcohol modification studies.

Physicochemical Properties	Parameters
Molar ratio o–cresol–furfural–formaldehyde	1:0.65:0.65
Reaction temperature, °C (1st stage/2nd stage)	120/90
Reaction time, hours (1st stage/2nd stage)	11/1
Free o–cresol content, wt.%	2.2
Free furfural content, wt.%	0.2
Free formaldehyde content, wt.%	<0.1
Molecular weight, g/mol	1245
Glass transition temperature, °C	207
Non-volatile mattercontent, wt.%	98
Physical state	Solid

**Table 4:** Signals assigned based on one-dimensional spectra.

Functional Group	^13^C NMR		^1^H NMR	
	Chemical Shift δ [ppm]	Symbol	Chemical Shift δ [ppm]	Symbol
–CH_3,_ –CH_2_-	29.35–30.46	C-7, C-19, K–CH_2_–K	2.08–2.56	C_7_–H_3_, C_19_–H_3_
–CH–	43.79–43.98; 44.40–44.80; 45.20–45.75	C-8	2.08–2.56	C_8_–H
–CH_2_–O–	85.72–85.85	K–CH_2_–O–CH_2_–K	4.37–4.52	K–CH_2_–O–CH_2_–K
–CH_2_–OH	92.30; 92.40	C-20	4.68–4.83	C_20_–H_a,b_
–CH_2_–O–	92.97–93.70	F–CH_2_–O– F–CH_2_–O–H	3.57–3.70	F–CH_2_–O– F–CH_2_–O–H
–CH– in F	136.80–137.62, 139.66	C-11, C-12	5.68–6.45	C_11_–H;C_12_–H
–CH– in K	144.01, 144.11	C-3, C-5, C-16, C-18	6.80 i 6.88	C_3_–H;C_5_–H; C_16_–H;C_18_–H
–C– in K	146.83	C-1, C-2, C-4, C-6, C-13, C-14, C-15, C-16	—	—
–CH– in K	149.27, 150.80, 152.03, 154.66, 154.74, 156.20, 156.31, 156.86, 156.98, 158.70, 159.36, 159.43, 160.34, 160.29, 161.76	C-3, C-5, C-16, C-18	6.50–7.21	C_3_–H;C_5_–H; C_16_–H;C_18_–H
–C–	153.64, 153.69, 159.60	–C– from furfural	—	—
–CH– in F	163.39	C-11, C-12 (from a molecule containing F–CH_2_–O– and F–CH_2_–O–H)	6.23–6.35	
–CH– in F	171.23	C–10	7.25–7.50	C_10_–H

K is o–cresol and F is furfural.

**Table 5:** Conditions for the etherification reaction of o–cresol–furfural–formaldehyde resin with n-butanol.

Unnmodifiedresin, wt.%	20
n-butanol, wt.%	80
Acidic environment, pH	3
Reaction temperature, °C	110
Reaction time, hour	20

**Table 6:** Physicochemical properties of o–cresol–furfural–formaldehyde resin etherified with n-butanol.

Physicochemical Properties	Parameters	
Catalyst	Malonic Acid	Oxalic Acid
Free o–cresol content, wt.%	<1.0	<1.0
Free furfural content, wt.%	<1.0	<1.0
Free formaldehyde content, wt.%	<0.1	<0.1
Molecular weight, g/mol	3402	11,389
Glass transition temperature, °C	55	53
Non-volatile matter content, wt.%	17.52	19.91
Viscosity 20 °C, mPa·s	22.5	12.1

**Table 7:** Conditions for the etherification reaction of o–cresol–furfural–formaldehyde resin with 2-ethylhexanol.

Unmodified resin, wt.%	20
2-ethylhexanol, wt.%	80
Acidic environment, pH	2.5
Reaction temperature, °C	110
Reaction time, hour	20

**Table 8:** Physicochemical properties of o–cresol–furfural–formaldehyde resin etherified with 2-ethylhexanol.

Physical and Chemical Properties	Parameters	
Catalyst	Malonic Acid	Oxalic Acid
Free o–cresol content, wt.%	<1.0	<1.0
Free furfural content, wt.%	<0.1	<0.1
Free formaldehyde content, wt.%	<0.1	<0.1
Molecular weight, g/mol	6394	8334
Glass transition temperature, °C	48	53
Non-volatile matter content, wt.%	20.57	19.91
Viscosity 20 °C, mPa·s	248	12.1

**Table 9:** Properties of coating obtained from o–cresol–furfural–formaldehyde resin.

Physico-Mechanical Properties	o–Cresol–Furfural–Formaldehyde Resin Modified with	
	n-Butanol	2-Ethylhexanol
Thickness ^a^ (dried coating)µm	18 ± 3	20 ± 3
Flexibility ^b^ (mm)	6	4
Cross-cut adhesion ^c^ (grades)	1	0
Hardness ^d^	0.89	0.81

^a^ Thickness according to EN ISO 2808:1999 [32], ^b^ Flexibility by bend test (conical mandrel) according to EN ISO 4618:2014 [33], ^c^ Cross-cut adhesion according to EN ISO 2409:2013 [34], ^d^ Hardness by pendulum dumping test according to EN ISO 1522:2008 [35].

## Data Availability

The original contributions presented in the study are included in the article, further inquiries can be directed to the corresponding author.

## References

[1] Rivero G., Fasce L.A., Cerè S.M., Manfredi L.B. (2014). Furan resins as replacement of phenolic protective coatings: Structural, mechanical and functional characterization. Prog. Org. Coat..

[2] Mougel C., Garnier T., Cassagnau P., Sintes-Zydowicz N. (2019). Phenolic foams: A review of mechanical properties, fire resistance and new trends in phenol substitution. Polymer.

[3] Oliveira F.B., Gardrat C., Enjalbal C., Frollini E., Castellan A. (2008). Phenol–Furfural Resins to Elaborate Composites Reinforced with Sisal Fibers––Molecular Analysis of Resin and Properties of Composites. J. Appl. Polym. Sci..

[4] Patel A.U., Soni S.S., Patel H.S. (2009). Synthesis, Characterization and Curing of o–cresol–Furfural Resins. Int. J. Polym. Mater..

[5] Hirano K., Asami M. (2013). Phenolic resins—100 years of progress and their future. React. Funct. Polym..

[6] Pilato L. (2013). Phenolic resins: 100 Years and still going strong. React. Funct. Polym..

[7] Tang K., Zhang A., Ge T., Liu X., Tang X., Li Y. (2021). Research progress on modification of phenolic resin. Mater. Today Commun..

[8] Sarika P.R. (2020). Bio-BasedAlternatives to Phenol and Formaldehyde for the Production of Resins. Polymers.

[9] Win D.T. (2005). Furfural—Gold from Garbage. Au J. Technol..

[10] Fink J.K. (2005). Reactive Polymers Fundamentals and Applications.

[11] Depta M., Jaszcz K. (2020). Furfural as an alternative for formaldehyde in production of phenolic resins. Przemysł Chem..

[12] Cardarelli F. (2018). A Concise Desktop Reference.

[13] Ahujaa S., Singh D. (2011). A kinetic model of alkali catalyzed phenol-furfural novalac resinification. Polym. Polym. Compos..

[14] Liu J., Xuan D., Chai J., Guo D., Huang Y., Liu S., Chew Y.T., Li S., Zheng Z. (2020). Synthesis and thermal properties of resorcinol–furfural thermosetting resin. ACS Omega.

[15] Asaro L., Seoane I.T., Fasce L.A., Cyras V.P., Manfredi L.B. (2019). Development of low environmental impact protective coatings based on a furan resin and cellulose nanocrystals. Prog. Org. Coat..

[16] Liu F., Yang L., Huang Y., Jiang P., Li G., Jiang W., Liu X., Fan Z. (2017). Performance of resin bonded sand for magnesium alloy casting. J. Manuf. Process..

[17] Nakamura K., Okuyama K., Takase T. (2017). Magnetic properties of magnetic glass-like carbon prepared from furan resin alloyed with magnetic fluid. J. Magn. Magn. Mater..

[18] Rivero G., Pettarin V., Vázquez A., Manfredi L.B. (2011). Curing kinetics of a furan resin and its nanocomposites. Thermochim. Acta.

[19] Vergara U., Sarrionandia M., Gondra K., Aurrekoetxea J. (2014). Polymerization and curing kinetics of furan resins under conventional and microwave heating. Thermochim. Acta.

[20] Albert D.F., Andrews G.R., Mendenhall R.S., Bruno J.W. (2001). Supercritical mathanol drying as a convenient route to phenolic-furfural aerogels. J. Non-Cryst. Solids.

[21] Teranishi Y., Kobayashi T., Yasuda E., Iwaki M., Kakihana M., Fukushima M., Nakamura K., Tanabe Y. (2005). Radiation damages and bubble formation of ion implanted furan-resin-derived carbon. Surf. Coat. Technol..

[22] Teranishi Y., Tanabe Y., Kobayashi T., Nakamura K., Fukushima M., Ishizuka M., Mitsuo A., Uematsu T., Isao N., Shimizu K. (2009). Graphitization behavior of the implanted furan-resin-derived carbon. Nucl. Instrum. Methods Phys. Res. B.

[23] Cheng Y., Sui G., Liu H., Wang X., Yang X., Wang Z. (2019). Preparation of highly phenol substituted bio-oil–phenol–formaldehyde adhesives with enhanced bonding performance using furfural as crosslinking agent. J. Appl. Polym. Sci..

[24] Ugryumov S.A., Patrakov R.V. (2011). The Use of Furan Oligomers for Modifying Phenol Formaldehyde Resin in Plywood Industry. Polym. Sci..

[25] Żmihorska-Gotfryd A. (2000). Preparation and properties of glycol-and polyetherol-modified phenol-formaldehyde resins. Polimery.

[26] Knop A., Pilato L.A. (1985). Phenolic Resins.

[27] (1995). Plastics—Phenolic Resins—Determination of Free-Formaldehyde Content—Hydroxylamine Hydrochloride Method.

[28] (1995). Plastics-Liquid Phenolic Resins—Conventional Determination of Non-Volatile Matter.

[29] (1986). Epoxy Resins—Methods of Testing—Determination of Viscosity.

[30] (1996). Molecular Absorption Spectrometry.

[31] Ishida S., Wakaki S., Kato Y., Nakamoto Y. (1984). Computational studies of reactions of phenols with aldehydes. J. Ind. Eng. Chem..

[32] (1999). Paints and Varnishes—Determination of Film Thickness.

[33] (2006). Paints and Varnishes—Bend Test (Conical Mandrel).

[34] (2013). Paints and Varnishes—Cross-Cut Adhesion.

[35] (2008). Paints and Varnishes—Hardness by Pendulum Dumping Test.

[36] Żmihorska-Gotfryd A. (2004). Coating compositions based on modified phenol-formaldehyde resin and urethane prepolymers. Prog. Org. Coat..

[37] Regulation E. (2008). 1272/2008,‘Regulation (EC) No 1272/2008 of the European Parliament and of the Council of 16 December 2008 on classification, labelling and packaging of substances and mixtures, amending and repealing Directives 67/548/EEC and 1999/45/EC, and amending Regulation (EC) No 1907/2006. Off. J. Eur. Union.

